# Riesgo de sesgo de publicación en intervenciones terapéuticas para la COVID-19

**DOI:** 10.26633/RPSP.2021.157

**Published:** 2021-12-16

**Authors:** Santiago Hasdeu, Fernando Tortosa

**Affiliations:** 1 Red Argentina Pública de Evaluación de Tecnologías Sanitarias Argentina; 2 Ministerio de Salud de la Provincia de Río Negro Argentina

**Keywords:** Metaanálisis como asunto, revisión sistemática, sesgo de publicación, COVID-19, evaluación de la tecnología biomédica., Meta-analysis as topic, systematic review, publication bias, COVID-19, technology assessment, biomedical, Metanálise como asunto, revisão sistemática, viés de publicação, COVID-19, avaliação da tecnologia biomédica.

## Abstract

En este artículo se describe el sesgo de publicación, sus causas más frecuentes, sus características, las herramientas regulatorias para evitarlo y algunas técnicas estadísticas para analizarlo. Se explican y aplican estas técnicas a tres intervenciones terapéuticas relacionadas con la enfermedad por el coronavirus 2019 (COVID-19, por su sigla en inglés): corticoides, ivermectina y tocilizumab; se detectó riesgo de sesgo de publicación para ivermectina y tocilizumab. Las revisiones sistemáticas y los metaanálisis son diseños de investigación secundaria que constituyen una referencia para guiar la toma de decisiones. Son propensos a distintos tipos de sesgo, que es una desviación sistemática en los resultados. Aun desarrollados con rigor metodológico, su validez puede verse amenazada por el sesgo de publicación. Este se define como el acto de ocultar o retrasar la publicación, retener datos surgidos de los estudios de investigación, o ambos. Hasta la mitad de los ensayos controlados que se realizan permanecen sin publicarse. Durante la pandemia por virus H1N1, el sesgo de publicación de estudios financiados por la industria llevó a recomendar y comprar en gran escala el fármaco oseltamivir que, luego se supo, no tenía efectos beneficiosos relevantes. Dos tercios del financiamiento de los estudios clínicos para COVID-19 provienen de la industria farmacéutica. En el contexto de la pandemia actual por COVID-19, se publican estudios a un ritmo acelerado, por lo que resulta de gran trascendencia conocer e identificar el sesgo de publicación. Para reducir el sesgo de publicación es necesario regular el registro y la publicación de ensayos clínicos, pero esto requiere una coordinación de los países y organismos internacionales. Es importante sospechar e intentar identificar el sesgo de publicación para la toma de decisiones.

## Abstract (en)

This article describes publication bias, its most frequent causes, its characteristics, the regulatory tools to avoid it, and some statistical techniques to analyze it. These techniques are explained and applied to three therapeutic interventions related to the 2019 coronavirus disease (COVID-19): corticosteroids, ivermectin, and tocilizumab. Risk of publication bias was detected for ivermectin and tocilizumab. Systematic reviews and meta-analyses are secondary research designs that provide a reference to guide decision-making. They are prone to different types of bias, i.e., a systematic deviation in the results. Even if carried out with methodological rigor, their validity can be threatened by publication bias. This is defined as the act of concealing or delaying publication, withholding data arising from research studies, or both. Up to half of controlled trials remain unpublished. During the H1N1 virus pandemic, publication bias from industry-funded studies led to the recommendation and large-scale procurement of oseltamivir, a drug that later proved to have no relevant beneficial effects. Two-thirds of clinical trial funding for COVID-19 comes from the pharmaceutical industry. In the context of the COVID-19 pandemic, studies are published at an accelerated pace, making it very important to understand and identify publication bias. To reduce publication bias it is necessary to regulate the registration and publication of clinical trials, but this requires coordination among countries and international bodies. It is important to suspect and attempt to identify publication bias for decision making.

## RESUMO

Este artigo descreve o viés de publicação, suas causas mais frequentes, suas características, as ferramentas regulatórias para evitá-lo e algumas técnicas estatísticas para analisá-lo. Essas técnicas são explicadas e aplicadas a três intervenções terapêuticas relacionadas à doença pelo coronavírus 2019 (COVID-19, na sigla em inglês): corticoides, ivermectina e tocilizumabe. Detectou-se risco de viés de publicação para ivermectina e tocilizumabe. As revisões sistemáticas e as meta-análises são delineamentos de pesquisa secundária que constituem uma referência para orientar a tomada de decisão. Estão propensas a diferentes tipos de viés, que é um desvio sistemático nos resultados. Mesmo tendo sido desenvolvidas com rigor metodológico, sua validade pode ser ameaçada pelo viés de publicação, que é definido como o ato de ocultar ou atrasar a publicação, reter dados de pesquisa ou ambos. Até metade dos estudos controlados realizados jamais são publicados. Durante a pandemia pelo vírus H1N1, o viés de publicação de estudos financiados pela indústria levou à recomendação e à compra, em grande escala, do medicamento oseltamivir que, mais tarde, ficou conhecido por não ter efeitos benéficos relevantes. Dois terços do financiamento para os ensaios clínicos referentes à COVID-19 vêm da indústria farmacêutica. No contexto da atual pandemia de COVID-19, os estudos vêm sendo publicados em ritmo acelerado; portanto, é fundamental conhecer e identificar o viés de publicação. Para reduzi-lo, é necessário regulamentar o registro de a publicação de ensaios clínicos, o que requer coordenação entre países e organismos internacionais. É importante suspeitar e tentar identificar o viés de publicação para a tomada de decisão.

Las revisiones sistemáticas (RS) y los metaanálisis (MA) representan el más alto nivel de evidencia sobre intervenciones terapéuticas. Si están elaborados y actualizados de manera adecuada, se presentan como la fuente de información con menor riesgo de sesgos acerca de la eficacia de los tratamientos (1). Una RS tiene como objetivo reunir toda la evidencia empírica que cumple con criterios de elegibilidad previamente establecidos, con el fin de responder una pregunta específica de investigación. Utiliza métodos sistemáticos y explícitos, con el fin de minimizar sesgos, de esta manera, aporta resultados más fiables a partir de los cuales se puedan extraer conclusiones y tomar decisiones (2). El MA consiste en la aplicación de métodos estadísticos para resumir los resultados de estudios independientes (1). Al combinar la información de todos los estudios relevantes, el MA puede obtener estimaciones más precisas de los efectos que las derivadas de los estudios individuales. También permite investigar la consistencia de la evidencia y explorar diferencias entre estudios.

Las RS y MA se utilizan como insumo para elaborar guías de práctica clínica, confeccionar listados de medicamentos esenciales y para incorporar sus resultados en las evaluaciones económicas.

Las RS y los MA pueden verse afectados por distintos tipos de sesgos. Un sesgo es un error o desviación sistemática en los resultados (1). Existen múltiples sesgos, y comprenderlos es relevante porque existen en todo tipo de investigación y son difíciles de eliminar. Además, el sesgo puede ocurrir en cada etapa del proceso de investigación y tiene efecto en la validez y confiabilidad de los hallazgos del estudio. Una interpretación incorrecta de los datos puede tener consecuencias importantes en el sistema de salud (3). Entre los diferentes tipos de sesgos, uno de gran trascendencia es el sesgo de publicación, donde solo un subconjunto sesgado de todos los datos relevantes está disponible (4), ya sea porque se retrasó la publicación o porque se retuvieron datos (5).

Los estudios no identificados pueden producir estimaciones mayores o menores en relación con los efectos identificados. Es más frecuente el problema de los estudios "negativos", donde su omisión conduce a un sesgo que sobreestima el efecto de la intervención. Existen diferentes causas de sesgo de publicación, incluidos los que se relacionan con intereses financieros, el idioma de la publicación, los resultados del estudio, y el sesgo de familiaridad, de disponibilidad y de costos (6). Una revisión sistemática de la colaboración Cochrane ha identificado mayor riesgo de sesgo de publicación en estudios financiados por la industria farmacéutica en comparación con otras fuentes de financiamiento (7). La publicación de documentos internos como resultado de acuerdos de conciliación en litigios han revelado ejemplos de manipulación de la industria farmacéutica en la realización y publicación de estudios (8). Los resultados negativos se anuncian con menos frecuencia y demoran más tiempo que los resultados positivos en publicarse (9).

En este artículo se describen diferentes aspectos relacionados con el riesgo de sesgo de publicación en las RS y MA, sus características, las causas más frecuentes, las herramientas regulatorias para evitarlo y las técnicas estadísticas más utilizadas para analizarlo. Como ejemplo se realiza un análisis estadístico para valorar el riesgo de sesgo de publicación en tres intervenciones terapéuticas relacionadas con la enfermedad por el coronavirus 2019 (COVID-19, por su sigla en inglés).

## MAGNITUD Y EFECTO DEL SESGO DE PUBLICACIÓN

Las consecuencias del sesgo de publicación pueden ser muy graves, incluida la sobrestimación de los efectos de los tratamientos, la subestimación de los eventos adversos o el refuerzo de creencias en teorías inválidas (7). Se estima que aproximadamente 50% de los estudios realizados se mantienen ocultos (10). La influencia de la industria farmacéutica debe ser mencionada, ya que financia la mayoría de los estudios controlados aleatorizados (ECA) publicados. Diversas investigaciones observaron una mayor tendencia a que los ECA financiados con resultados negativos se mantengan sin publicar, en comparación con estudios independientes de la industria que encuentren resultados negativos (10). Una encuesta en facultades de medicina en Estados Unidos de América mostró que solo 1% de los investigadores tuvieron acceso a los datos completos del ECA, y solo 40% tenía control sobre la publicación (11). Así, los investigadores tienen poca o ninguna injerencia en el diseño de los ensayos y en el acceso a los datos crudos, y tienen una participación limitada en la interpretación de los datos.

## HISTORIA RECIENTE DEL SESGO DE PUBLICACIÓN EN LA PANDEMIA POR VIRUS H1N1

El caso del oseltamivir en la pandemia del virus influenza H1N1 es un ejemplo del gran efecto negativo de este tipo de sesgo, que debemos tener presente ante la actual pandemia por COVID-19 (12). En el momento en que se tomó la decisión, 60% de los datos de ensayos sobre este fármaco seguían sin publicarse. La Colaboración Cochrane había concluido que oseltamivir reducía el riesgo de neumonía, con base en un análisis combinado, elaborado por el fabricante, de 10 ensayos financiados por la industria. Ocho de estos eran estudios no publicados. El equipo Cochrane solicitó a los autores de cada estudio los datos primarios para analizarlos, pero no logró obtenerlos, sino hasta muchos años más tarde, luego de expirada la patente (12). Aún sin contar con todos los resultados solicitados, se publicó la RS, en la que se informaba un beneficio clínico con el uso de oseltamivir. Este fue uno de los insumos considerados para que la Organización Mundial de la Salud (OMS) decidiera incorporar el oseltamivir en su listado de medicamentos esenciales, y a una compra en gran escala por muchos países del mundo. La mayoría de los ensayos de tratamiento de fase III del laboratorio seguían sin publicarse una década después de su finalización (13). Este sesgo tuvo un efecto muy importante en la salud pública, distrajo recursos limitados y generó expectativas en el personal de salud y en la población. En un nuevo MA publicado en 2013, se encontró que oseltamivir reduce en 20 horas promedio la duración de los síntomas y ninguna prueba de reducción del riesgo de neumonía, ingreso hospitalario ni complicaciones que requirieron la administración de un antibiótico. La nueva revisión Cochrane confirmó estos resultados y dio más información sobre efectos indeseados como náuseas, vómitos y problemas mentales (13).

La ausencia de la publicación de datos imposibilita la evaluación adecuada de desenlaces como la eficacia, los efectos adversos y la mortalidad, lo que puede generar que, en ocasiones, las decisiones basadas en la evidencia no sean las decisiones óptimas (14). Conocer las características del riesgo de sesgo de publicación y el efecto que tuvo en la historia reciente de pandemia por virus H1N1 puede ser de gran importancia ante la actual pandemia por COVID-19.

## EL RIESGO DE SESGO DE PUBLICACIÓN EN EL CONTEXTO DE LA ACTUAL PANDEMIA

En el contexto de la pandemia por COVID-19, hay diversos factores que alertan sobre la potencial existencia de sesgos de publicación:

- Ante la aparición de este virus y enfermedad nuevos, en solo un año y medio se han publicado cientos de miles de artículos científicos, de diversa calidad metodológica. Se ha incrementado la utilización de artículos aún sin publicar (*preprints*), que representan una forma acelerada de dar a conocer investigaciones científicas que aún no cuentan con revisión de pares (15). Si bien esto ha sido muy valioso para avanzar en la diseminación de información relevante, también tiene algunos riesgos potenciales (9,16).
- Pese a la existencia de investigaciones impulsadas por los países, centros académicos y la OMS, como el caso del estudio Solidarity, dos tercios del financiamiento de los estudios clínicos para la COVID-19 provienen de la industria farmacéutica (17).
- Intereses de mercado: una intervención para la COVID-19 contaría con una enorme cantidad de potenciales usuarios, por lo que la pandemia representa una oportunidad única para los fabricantes de tecnologías de la salud. Ante la mencionada demanda y las limitaciones para facilitar el acceso a todos los potenciales usuarios, los precios de venta de las tecnologías presentan enormes incrementos, variaciones y distorsiones (18).
- Intereses geopolíticos: más allá del interés de los laboratorios, muchos países y bloques han mostrado comportamientos de defensa cerrada de las tecnologías producidas en sus países, con un efecto negativo en la investigación (19).
- Los decisores se encuentran presionados por la urgencia, la escasez de recursos y la sociedad que exige resultados sanitarios.

## UNA MIRADA CRÍTICA

Los antecedentes mencionados, sumados al contexto actual, deben llevarnos a tener una actitud crítica, de “sano escepticismo” (20) y una elevada sospecha de posible existencia de sesgo de publicación, especialmente ante nuevas indicaciones y tecnologías costosas protegidas por patentes.

## VÍAS REGULATORIAS PARA EVITAR EL SESGO DE PUBLICACIÓN

Existen herramientas regulatorias para evitar este sesgo, como las iniciativas de la Organización Panamericana de la Salud (OPS) para otorgar un número de identificación a los ECA (21) y para llevar un registro del grado de avance de cada uno de los países (22), iniciativas de OPS sobre las políticas de investigación (23-25), Open Data, All Trials (26), publicaciones conjuntas de revistas científicas y otros registros de estudios de investigación desde la etapa de protocolo, como ClinicalTrials, que buscan transparentar los resultados del mundo académico y, de esta manera, reducir el riesgo de sesgo de publicación (10). AllTrials es una iniciativa internacional de la Colaboración Cochrane, el British Medical Journal y otras instituciones, bajo la consigna “Todos los ensayos registrados, todos los resultados informados” (26). En la Región de las Américas, las diferencias existentes en las regulaciones sobre la transparencia en la investigación entre los distintos países se reflejan en diferencias en los sistemas de gobernanza de los comités de ética de la investigación (22). En algunos países, como es el caso de Brasil, la interrupción de la investigación y la falta de publicación deben explicarse al organismo nacional que los regula; esto no ocurre en todos los países, ni está establecido con claridad un mecanismo punitivo para desalentar que las conductas antiéticas se repitan (23). Pese a que la OMS recomienda (27), y la legislación de Estados Unidos y Europa exigen la publicación de resultados en línea dentro del año de completado un estudio, se constatan reiterados incumplimientos y prácticamente no se han aplicado sanciones (28).

## HERRAMIENTAS TÉCNICAS PARA REDUCIR EL RIESGO DE SESGO DE PUBLICACIÓN

En los MA, pueden aplicarse técnicas para reducir el riesgo de distorsiones por sesgo de publicación. Algunos de estos enfoques pertenecen a la búsqueda de estudios, mientras que otros son métodos estadísticos. Se recomienda a los autores de MA utilizar de forma sistemática una serie de técnicas para identificar ensayos no publicados: búsquedas en registros de ensayos, y contactos con investigadores y fabricantes (29).

El enfoque más confiable para la síntesis de evidencia en los MA es utilizar los datos de participantes individuales (30), pero esto no garantiza que estén libres de sesgos.

## HERRAMIENTAS ESTADÍSTICAS PARA DETECTAR RIESGO DE SESGO DE PUBLICACIÓN

### Interpretación de gráficos de embudo (*funnel plot*) y pruebas estadísticas

Existen varios métodos de efectos de estudios pequeños para evaluar y corregir el sesgo de publicación en los MA (31). Desde un punto de vista estadístico, estos poseen un alto error estándar (EE). Los métodos de efectos de estudios pequeños asumen que los estudios pequeños tienen más probabilidades de tener sesgo de publicación.

El modo habitual de inspeccionar los efectos de estudios pequeños es a través de los gráficos de embudo. Un gráfico de embudo es un gráfico de dispersión de los tamaños de efecto observados de los estudios en el eje X contra una medida de su ES en el eje Y. Por lo general, en los gráficos de embudo, el eje Y está invertido (lo que significa que los valores “más altos” en este eje representan EE más bajos). Cuando no hay sesgo de publicación, los puntos de datos en estos gráficos deben formar un embudo invertido aproximadamente simétrico, de allí su denominación (30). Los estudios en la parte superior del gráfico (aquellos con EE bajos) deben estar muy juntos y no muy lejos del tamaño del efecto agrupado. En la parte inferior del gráfico, con EE crecientes, el embudo “se abre” y se espera que los tamaños de los efectos se dispersen más hacia la izquierda y a la derecha del efecto agrupado.

El EE indica la precisión de un estudio: con el EE decreciente, se espera que el tamaño del efecto observado se convierta en un estimador cada vez mejor del tamaño del efecto real. Para facilitar la interpretación, la trama también incluye la forma de embudo idealizada que se espera que sigan los estudios. La línea vertical en el medio del embudo muestra el tamaño medio del efecto.

El gráfico con contornos permite una interpretación visual de la significancia estadística de los estudios. Estudios con grandes EE pueden caer en zonas de significancia estadística relevante, y viceversa.

El tamaño de la intersección de regresión brinda información sobre la asimetría en el gráfico de embudo. En cada modelo de regresión lineal, la intersección representa el valor de Y cuando todos los demás predictores son cero. El predictor en el modelo aquí presentado es la precisión de un estudio, por lo que la intersección muestra el puntaje esperado cuando la precisión es cero (es decir, cuando el error estándar de un estudio es infinitamente grande).

Cuando no hay sesgo de publicación, la puntuación debe estar esparcida alrededor de cero. Sin embargo, cuando el gráfico de embudo es asimétrico, por ejemplo, debido al sesgo de publicación, se espera que los estudios pequeños con tamaños de efecto muy altos estén considerablemente sobrerrepresentados en los datos, lo que lleva a un número sorprendentemente alto de estudios de baja precisión con valores mayores o iguales a 1,96; esto resultará en una intersección significativa.

Otra forma de calcular una estimación del tamaño del efecto ajustado es realizar un MA de límites de Rücker (31). Se tiene en cuenta el hecho de que los tamaños del efecto y los EE de los estudios no son independientes cuando hay efectos de estudios pequeños.

### Métodos de estudio de riesgo de sesgo en tres intervenciones para COVID-19

Se realizó un análisis del riesgo de sesgo de publicación de tres intervenciones terapéuticas relacionadas con COVID-19 en las que exista un efecto beneficios: esteroides sistémicos, tocilizumab e ivermectina (esta última con baja certeza en los efectos de la intervención) (33). Se realizó una búsqueda de ECA que valorasen intervenciones incluidos en RS vivas. Los estudios incluidos y sus efectos corresponden a los que se encuentran incluidos en dichas revisiones hasta el día 1 de junio de 2021 (33,34). Para el análisis de estudios no publicados y ensayos en curso registrados, también se buscó en medRxiv, bioRxiv, arXiv, International Clinical Trials, plataforma de registro (http://apps.who.int/trialsearch), plataforma Epistemonikos COVID-19 LOVE (http://app.iloveevidence.com/loves/) y ClinicalTrails.gov (http://clinicaltrials.gov/).

Para cada intervención analizada se realizó un gráfico de embudo y análisis estadístico del riesgo de sesgo de publicación: asimetría en el gráfico de embudo de contornos resaltados, prueba de regresión lineal, prueba de Eggers y metaanálisis de límites de Rücker. Los cálculos y gráficos estadísticos se realizaron en el *software* R^®^.

## RESULTADOS DEL ESTUDIO DE RIESGO DE SESGO EN TRES INTERVENCIONES PARA COVID-19

### Esteroides sistémicos

**FIGURA 1.**
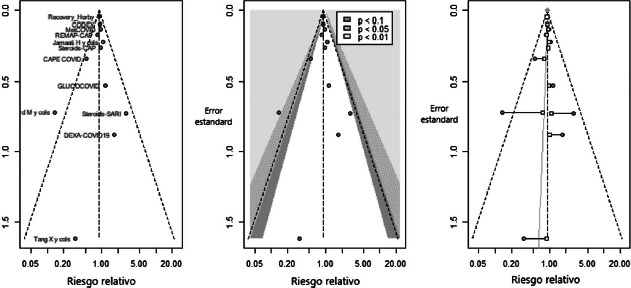
Gráfico de embudo, de contornos y de límites del metaanálisis para esteroides sistémicos

En el caso de los esteroides sistémicos, en el gráfico de embudo (figura 1) se observa que los estudios se distribuyen alrededor de la estimación media del efecto de un modo simétrico, en forma de un embudo invertido. Esta simetría se percibe en forma más clara cuando se la observa en el gráfico de contornos (los límites en las estimaciones de efectos de la mayoría de los estudios se encuentran por debajo del área del embudo).

**CUADRO 1.** Prueba de regresión lineal para detectar asimetría del gráfico de embudo y prueba de Eggers para esteroides sistémicos, tolcilizumab e ivermectina

Prueba estadística	Esteroides sistémicos	Tocilizumab	Ivermectina
Prueba de regresión lineal y prueba de Eggers	-0,3	2,61	-1,97
Valor de P	0,78	0, 04	0,08
Error estándar	0,47	0,38	0,72
Riesgo de sesgo	Bajo	No puede descartarse	No puede descartarse

***Fuente*:** elaboración propia.

**CUADRO 2.** Ensayos clínicos aleatorizados registrados, publicados y sin datos disponibles sobre intervenciones farmacológicas durante la pandemia de COVID-19

Tipo de ensayo clínico	Esteroides	Tocilizumab	Ivermectina
Registrados	200	108	139
Prepublicados y publicados	15	10	11
Sin datos disponibles (%)	115 (57)	81 (75)	88 (63)

***Fuente*:** elaboración propia.

***Fuente*:** elaboración propia.

En el cuadro 1 se muestran las pruebas estadísticas de regresión y de Eggers, y corroboran la simetría de la distribución de los efectos de los estudios observada en el gráfico de embudo. Como se observa en el cuadro 2, se encuentran registrados 200 ensayos clínicos relacionados con el uso de esteroides sistémicos, de los cuales 15 se encuentran publicados o prepublicados, mientras que 115 (57%) no ofrecen datos. La sospecha de sesgo de publicación es baja en el caso de los esteroides.

### Ivermectina

**FIGURA 2.**
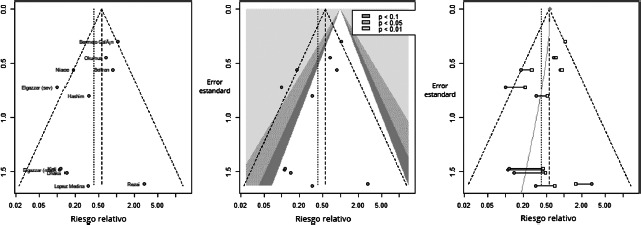
Gráfico de embudo, de contornos y de límites del metaanálisis para ivermectina

El gráfico de embudo en la figura 2 muestra una distribución asimétrica de los efectos, con una agrupación de varios estudios pequeños y con menor precisión, ubicados en la base del embudo y en la zona de efecto beneficioso muy significativo (zona de riesgo relativo [RR] menor de 1). Los estudios más precisos, en este caso 3 (tres) con intervalos de confianza angostos, se ubican en la punta del gráfico y en el área de un RR mayor de 1.

Las pruebas estadísticas para detectar asimetría muestran una intersección que se aleja significativamente del 0 (cuadro 1). La distribución asimétrica de las estimaciones de efecto que puede observarse en los gráficos de embudo se debe a los estudios pequeños de baja precisión, con amplios intervalos de confianza mostrando un efecto beneficioso, mientras que los estudios más precisos con límites angostos se ubican en el vértice de los gráficos. Las pruebas de regresión confirman la asimetría observada y hacen presumir que podría existir sesgo de publicación, en favor de estudios pequeños con resultados positivos con ivermectina y ausencia de estudios pequeños con resultados negativos. Se encuentran registrados 139 ensayos clínicos (cuadro 2), de los cuales 11 se encuentran publicados o prepublicados, mientras que 88 (63%) no ofrecen datos.

*Fuente*: elaboración propia.

### Tocilizumab

**FIGURA 3.**
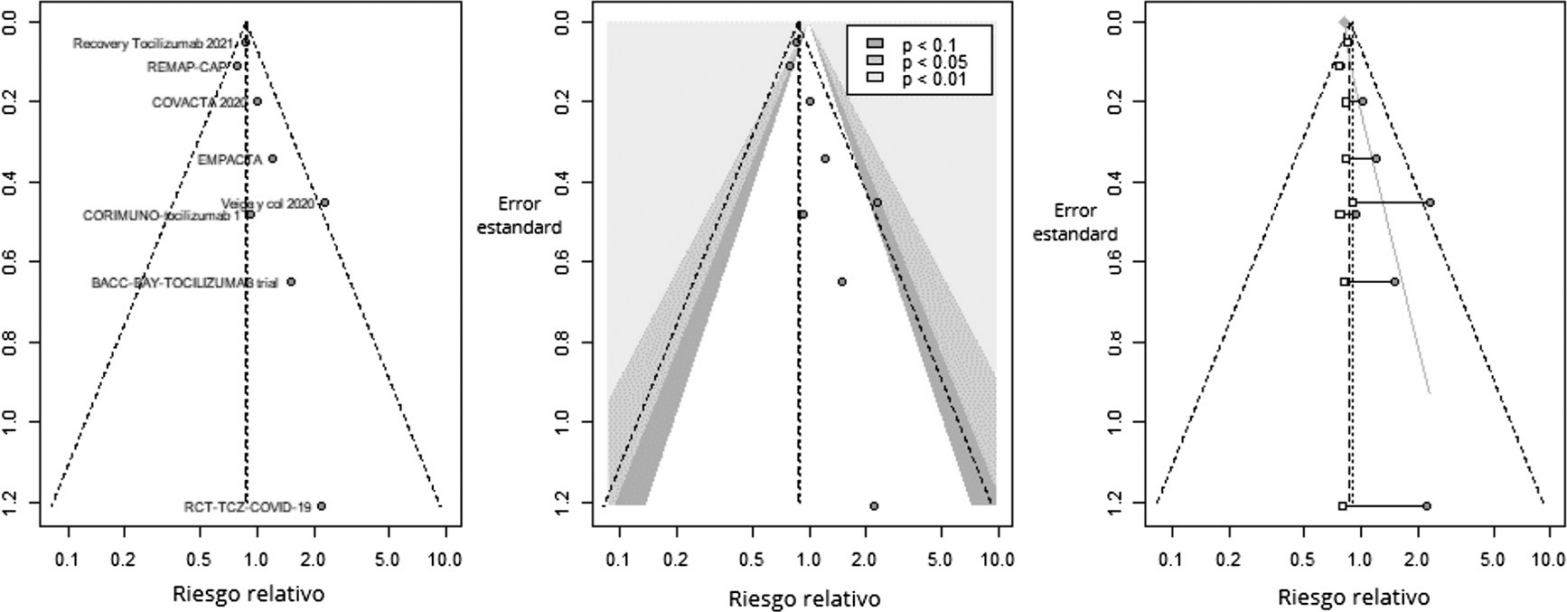
Gráfico de embudo, de contornos y de límites del metaanálisis para tocilizumab

La mayoría de los estudios presentan una estimación central del efecto alrededor de la media de este, como se observa en la figura 3. Los límites de los estudios más imprecisos y con errores estándares mayores, se encuentran alrededor del no efecto (RR mayor a la media del efecto) con uno de los estudios en el área no significativa de la gráfica de contornos. Los estudios más imprecisos (mayor EE) y con límites más amplios (mayor dispersión del efecto) se encuentran en el área del gráfico con ausencia de beneficio (mayor riesgo relativo). En este caso, los estudios con mayor número de pacientes y más precisos (n = 2) se encuentran en el vértice del embudo, pero en el límite de la significancia estadística.

Las pruebas de regresión lineal para detectar la asimetría del embudo y la prueba de Eggers mostraron una intersección diferente de cero, lo que confirma la asimetría de la distribución de los estudios. Se encuentran registrados 108 ensayos clínicos (cuadro 2), de los cuales 10 se encuentran publicados o prepublicados, mientras que 81 (75%) no ofrecen datos. En el caso del tocilizumab, no puede descartarse el sesgo de publicación.

En el cuadro 1 se presenta una comparación del riesgo de sesgo de publicación con el método de Eggers entre las tres tecnologías seleccionadas. En el caso de los esteroides sistémicos, donde no existe sospecha de sesgo de publicación, la intersección de la prueba es menor a 1, incluso cercana al cero. Esto no ocurre con las otras dos intervenciones en las cuales se sospecha exista riesgo de sesgo de publicación, donde la intersección de la prueba se aleja sustancialmente del cero.

*Fuente*: elaboración propia.

En el cuadro 2 se observa que, del total de los protocolos registrados, 6-8% fueron publicado o prepublicado, mientras que más de 60% de los estudios registrados no aporta datos luego de 6 meses de iniciado este registro.

## DISCUSIÓN

El sesgo de publicación representa una amenaza a la validez de las RS y los MA y, por extensión, a la toma de decisiones basadas en evidencias (1,2). Su frecuencia es tan elevada que se estima que hasta la mitad de los ensayos controlados se mantienen sin publicar (10). Esto ha provocado daños a la salud y enormes costos de oportunidad asignando recursos limitados a intervenciones ineficaces, como se describió en el caso del oseltamivir durante la pandemia por el virus influenza H1N1 (12).

Las vías normativas y regulatorias serían las adecuadas para evitarlo, pero esto requiere una articulación y colaboración internacional para presentar una estrategia homogénea y estricta que pueda desincentivar el sesgo de publicación (25). Diversas iniciativas de la OMS y la OPS han logrado avances a nivel mundial y en la Región (23,24), y hacen sinergia con registros internacionales de ensayos clínicos e iniciativas como la de AllTrials (26). Sin embargo, se constatan reiterados incumplimientos a las recomendaciones de la OMS y a la legislación vigente, que no se acompañan de sanciones (27,28).

Hasta que se cumpla con la obligación del registro de todo ensayo clínico, y la publicación a tiempo de todos los resultados de los ensayos realizados, solo existen algunas técnicas disponibles que permiten sospechar y detectar riesgo de sesgo de publicación en las RS y los MA (29). Se describen las estrategias de búsqueda exhaustivas, el análisis a partir de los datos individuales de los participantes de cada estudio, y pruebas estadísticas como el de Eggers y gráfico de embudo. Muchos estudios han evaluado el rendimiento de diferentes enfoques, y encontraron que ningún método de análisis supera sistemáticamente a los demás (30). A menudo, es difícil identificar qué enfoque se adapta mejor a los datos recopilados, y se desconoce el grado exacto en que la publicación selectiva ha afectado los resultados. Sin embargo, al aplicar varias técnicas combinadas destinadas a detectar el sesgo, se puede producir algo similar a una variedad de efectos verdaderos creíbles (29).

Los ejemplos provistos permiten observar cómo el MA sobre el tratamiento de la COVID-19 con corticoides sistémicos no parece sugerir la existencia de sesgo de publicación. Por otro lado, los ejemplos del análisis estadístico de los MA de ivermectina y tocilizumab hacen sospechar distintos tipos de sesgo de publicación.

La sospecha de sesgo de publicación debe orientar a un “sano escepticismo” al momento de interpretar las evidencias publicadas. Esto adquiere especial importancia para la toma de decisiones en el ámbito de la salud ante la actual situación de pandemia.

## Declaración.

Las opiniones expresadas en este manuscrito son responsabilidad del autor y no reflejan necesariamente los criterios ni la política de la *RPSP/PAJPH* y/o de la OPS.

## References

[1] 1. Lasserson TJ, Thomas J, Higgins JPT. Capítulo 1: Starting a review. En: Higgins JPT, Thomas J, Chandler J, Cumpston M, Li T, Page MJ, Welch VA (editors). Cochrane Handbook for Systematic Reviews of Interventions. Version 6.0. Cochrane Collaboration; 2019. Disponible en: http://www.training.cochrane.org/handbook

[2] 2. The Grading of Recommendations Assessment, Development and Evaluation (GRADE) approach. GRADE handbook. Disponible en: https://gdt.gradepro.org/app/handbook/handbook.html#h.ged5uqebmir9 Acceso el 21 de mayo de 2019.

[3] 3. Evidence-Based Nursing [Internet]. Bias in research. Disponible en: https://ebn.bmj.com/content/17/4/100.long Acceso el 23 de setiembre de 2021.

[4] 4. Rothstein HR, Sutton AJ, Borenstein M. Publication bias in meta-analysis: prevention, assessment and adjustments. Nueva Jersey: John Wiley & Sons; 2005.

[5] 5. Page MJ, Sterne JAC, Higgins JPT, Egger M. Investigating and dealing with publication bias and other reporting biases in meta-analyses of health research: a review. Research Synthesis Methods. 2021;12(2):248-59. 10.1002/jrsm.1468 33166064

[6] 6. Dechartres A, Altman DG, Trinquart L, Boutron I, Ravaud P. Association between analytic strategy and estimates of treatment outcomes in meta-analyses. JAMA. 2014;312(6):623-30. 10.1001/jama.2014.8166 25117131

[7] 7. Lundh A, Lexchin J, Mintzes B, Schroll JB, Bero L. Industry sponsorship and research outcome. Cochrane Database Syst Rev. 2017;2:MR000033. 10.1002/14651858.MR000033.pub3 PMC8132492 28207928

[8] 8. Spurgeon D. Report clears researcher who broke drug company agreement. BMJ. 2001;323:1085. 10.1136/bmj.323.7321.1085 PMC1121590 11701563

[9] 9. Qunaj L, Jain RH, Atoria CL, Gennarelli RL, Miller JE, Bach PB. Delays in the publication of important clinical trial findings in oncology. JAMA Oncology. 2018;4(7):e180264. Disponible en: 10.1001/jamaoncol.2018.0264 PMC6145729 29710325

[10] 10. Schmucker C, Schell LK, Portalupi S, Oeller P, Cabrera L, Bassler D, et al. Extent of non-publication in cohorts of studies approved by research ethics committees or included in trial registries. PloS one. 2014;9(12):e114023. 10.1371/journal.pone.0114023 PMC4275183 25536072

[11] 11. Schulman KA, Seils DM, Timbie JW, Sugarman J, Dame LA, Weinfurt KP, et al. A national survey of provisions in clinical-trial agreements between medical schools and industry sponsors. NEJM. 2002;347(17):1335-41. 10.1056/NEJMsa020349 12397192

[12] 12. The British Medical Journal. Tamiflu campaign [Internet]. Disponible en https://www.bmj.com/tamiflu

[13] 13. Jefferson T, Jones M, Doshi P, Spencer EA, Onakpoya I, Heneghan CJ. Oseltamivir for influenza in adults and children: systematic review of clinical study reports and summary of regulatory comments. BMJ. 2014;348:g2545. Doi:10.1136/bmj.g2545 10.1136/bmj.g2545 PMC3981975 24811411

[14] 14. Dwan K, Gamble C, Williamson PR, Kirkham JJ. Systematic review of the empirical evidence of study publication bias and outcome reporting bias — an updated review. PLOS ONE. 2013;8(7):e66844. Disponible en: https://journals.plos.org/plosone/article?id=10.1371/journal.pone.0066844 10.1371/journal.pone.0066844 PMC3702538 23861749

[15] 15. Mather, Nicole. How we accelerated clinical trials in the age of coronavirus. Nature. 2020;584(7821):326. 10.1038/d41586-020-02416-z 32812005

[16] 16. Brainard, J. New tools aim to tame pandemic paper tsunami. Science. 2020;368(6494):924-925. Doi: 10.1126/science.368.6494.924 10.1126/science.368.6494.924 32467369

[17] 17. Robinson JC. Funding of pharmaceutical innovation during and after the COVID-19 pandemic. JAMA. 2021;325(9):825-826. Doi:10.1001/jama.2020.25384 10.1001/jama.2020.25384 33443546

[18] 18. Organización Mundial de la Salud. Shortage of personal protective equipment endangering health workers worldwide. Ginebra: OMS; 2020. Disponible en: https://www.who.int/news-room/detail/03-03-2020-shortage-of-personal-protective-equipment-endangering-health-workers-worldwide

[19] 19. Nature Editorial. Protect precious scientific collaboration from geopolitics. Nature. 2021;593(477). Doi: 10.1038/d41586-021-01386-0 34040213

[20] 20. Moreno Rodríguez MA. La medicina basada en la evidencia y la práctica médica individual. Revista Cubana de Medicina. 2005;44:3-4.

[21] 21. Krleža-Jeriç K, Lemmens T, Reveiz L, Cuervo LG, Bero LA. Prospective registration and results disclosure of clinical trials in the Americas: a roadmap toward transparency. Rev Panam Salud Publica.2011;30:87-96. 22159656

[22] 22. Organización Panamericana de la Salud. Política de Investigación para la Salud. Documento CD49/10, 49.º Consejo Directivo, 61.ª sesión del Comité Regional de la OMS para las Américas. Washington D.C.: OPS; 2009. Disponible en: https://www.paho.org/hq/dmdocuments/2009/CD49-10-s.pdf

[23] 23. Rodríguez-Feria P, Cuervo LG. Progress in trial registration in Latin America and the Caribbean, 2007-2013. Rev Panam Salud Publica. 2017;41:e31. 10.26633/RPSP.2017.31 PMC6612714 31363353

[24] 24. Organización Mundial de la Salud. Pharmaceutical sector country profiles data and reports. Región de las Américas. Washington D.C.: OPS; 2010. Disponible en: www.who.int/medicines/areas/coordination/coordination_assessment/en/index1.html

[25] 25. Lemmens T, Herrera Vacaflor C. Transparencia sobre los ensayos clínicos en la Región de las Américas: necesidad de coordinar las esferas regulatorias. Rev Panam Salud Publica. 2019;43.

[26] 26. HeneghanC. Open letter to EMA from clinical trial participants. Disponible en: https://www.alltrials.net/news/open-letter-to-ema-fromclinical-trial-participants/ Acceso el 30 de mayo de 2021.

[27] 27. Moorthy VS, Karam G, Vannice KS, Kieny M-P. Rationale for WHO’s new position calling for prompt reporting and public disclosure of interventional clinical trial results. PLOS Medicine. 2015;12(4):e1001819. Disponible en: https://journals.plos.org/plosmedicine/article?id=10.1371/journal.pmed.1001819. 10.1371/journal.pmed.1001819 PMC4396122 25874642

[28] 28. DeVito NJ, Bacon S, Goldacre B. FDAAA TrialsTracker: a live informatics tool to monitor compliance with FDA requirements to report clinical trial results. 2019:266452. Disponible en: https://www.biorxiv.org/content/10.1101/266452v4

[29] 29. Ikhlaaq A, Sutton AJ, Riley RD. Assessment of publication bias, selection bias, and unavailable data in meta-analyses using individual participant data: a database survey. BMJ. 2012;344:d7762. Doi: 10.1136/bmj.d7762 (Published 03 January 2012) 22214758

[30] 30. Borenstein M, Hedges LV, Higgins JPT, Rothstein HR. Introduction to meta-analysis. Nueva Jersey: John Wiley & Sons; 2011.

[31] 31. Rücker G, Schwarzer G, Carpenter JR, Binder H, Schumacher M. Treatment-effect estimates adjusted for small-study effects via a limit meta-analysis. Biostatistics. 2011;12(1):122-142. Doi:10.1093/biostatistics/kxq046 10.1093/biostatistics/kxq046 20656692

[32] 32. Peters JL, Sutton AJ, Jones DR, Abrams KR, Rushton L. Comparison of two methods to detect publication bias in meta-analysis. JAMA 2006;295(6):676-80. 10.1001/jama.295.6.676 16467236

[33] 33. Organización Panamericana de la Salud. Ongoing living update of potential COVID-19 therapeutics options: summary of evidence: rapid review. Washington D.C.: OPS; 2021. Disponible en: https://iris.paho.org/handle/10665.2/52719 Acceso el 19 de mayo de 2021.

[34] 34. Siemieniuk RA, Bartoszko JJ, Ge L, Zeraatkar D, Izcovich A, Kum E, et al. Drug treatments for COVID-19: living systematic review and network meta-analysis. BMJ. 2020;370:m2980. 10.1136/bmj.m2980 PMC7390912 32732190

